# Polish adaptation and validation of the hip disability and osteoarthritis outcome score (HOOS) in osteoarthritis patients undergoing total hip replacement

**DOI:** 10.1186/s12955-020-01390-4

**Published:** 2020-05-12

**Authors:** Marek K. Gojło, Przemysław T. Paradowski

**Affiliations:** 1 Department of Orthopaedics and Traumatology, Ministry of Interior and Administration Hospital, Warmia and Mazury Oncology Centre, Olsztyn, Poland; 2 Division of Orthopaedics, Department of Surgical and Perioperative Sciences, Sunderby Research Unit, Umeå University, Umeå, Sweden; 3 Department of Orthopaedics and Traumatology, Faculty of Health Sciences, Ludwik Rydygier Collegium Medicum, Nicolaus Copernicus University in Toruń, Jan Biziel University Hospital, Bydgoszcz, Poland; 4 Clinical Epidemiology Unit, Orthopaedics, Department of Clinical Sciences, Lund University, Lund, Sweden

**Keywords:** Hip disability and osteoarthritis outcome score (HOOS), Osteoarthritis, Total hip replacement, Patient-relevant outcome, Cross-cultural adaptation, Psychometrics

## Abstract

**Background:**

The Hip disability and Osteoarthritis Outcome Score (HOOS) is a frequently used patient-reported outcome measure (PROM) for assessment of hip disorders and treatment effects following hip surgery. The objective of the study was to translate and adapt the Hip disability and Osteoarthritis Outcome Score (HOOS) into Polish and to investigate the psychometric properties of the HOOS in patients with osteoarthritis undergoing total hip replacement (THR).

**Materials and methods:**

The Polish version of the HOOS was developed according to current guidelines. Patients completed the HOOS, Short Form 36 Health Survey (SF-36), the visual analogue scale (VAS) for pain and the global perceived effect (GPE) scale. Psychometric properties including interpretability (floor/ceiling effects), internal consistency (Cronbach’s alpha), test-retest reliability (intra-class correlation coefficient, ICC), convergent construct validity (a priori hypothesized Spearman’s correlations between the HOOS subscales, the generic SF-36 measure and the VAS for pain) and responsiveness (effect size, association between the HOOS and GPE scores) were analyzed.

**Results:**

The study included 157 patients (mean age 66.8 years, 54% women). Floor effects were found prior to THR for the HOOS subscales Sports and Recreation and Quality of Life. The Cronbach’s alpha was over 0.7 for all subscales indicating satisfactory internal consistency. The test–retest reliability was good for the HOOS subscale Pain (0.82) and excellent for all other subscales with ICCs ranging from 0.91 to 0.96. The minimal detectable change ranged from 12.0 to 26.2 on an individual level and from 1.4 to 3.0 on a group level. Seven out of eight a priori hypotheses were confirmed indicating good construct validity. Responsiveness was high since the expected pattern of effect sizes in all subscales was found.

**Conclusions:**

The Polish version of the HOOS demonstrated good reliability, validity and responsiveness for use in patient groups having THR.

## Introduction

Assessment of pain and function in patients with osteoarthritis (OA) has become routine in both clinical practice and research. For patients with hip and knee OA, the Western Ontario and McMaster Universities Osteoarthritis Index (WOMAC) is still a highly recommended and frequently used patient-relevant outcome measure (PROM) [1]. However, since the WOMAC does not cover all important aspects of outcome, especially in subjects with higher physical demands, it has been further developed by completing available subscales and adding two new dimensions: Sport and Recreation Function and joint-related Quality of Life. The Knee Injury and Osteoarthritis Outcome Score (KOOS), an extension of the WOMAC, was initially constructed as a measure of PRO in studies of the treatment of anterior cruciate ligament and meniscus injury and later validated even for middle-aged patients with OA [2]. Another measure, the Hip disability and Osteoarthritis Outcome Score (HOOS) was adapted from the KOOS to be used in patients eligible for both, basic and surgical treatment of OA [3, 4].

The HOOS is a simple self-administered instrument that was originally developed in English and Swedish [3], and is currently available in 23 languages and language variants [5].

So far, there have been no available formally cross-culturally adapted PROs that could be used for assessment of functional status and quality of life following hip surgery in Poland. Thus, the objective of this study was 1) to linguistically and cross-culturally translate the HOOS into Polish and 2) to test its psychometric properties as expressed by reliability, validity and responsiveness of the Polish version of the HOOS in patients with end-stage hip OA who had undergone THR.

## Methods

### Linguistic and cross-cultural translation process

#### Translation of the questionnaire

The translation and cross-cultural adaptation of the HOOS from the source Swedish and English versions was performed according to the recommendations by Beaton et al. [6].

A total of five persons were involved in the translational process. Two independent forward translations (T1, T2) were performed from the English version by an orthopaedic surgeon, who was a native speaker of Polish and fluent in English and a professional translator. Another independent translation (T3) was performed from the Swedish version by a medical professional of Polish origin, fluent in Swedish. A final unified version of these three translations was reached after a consensus meeting. Then two native English-speaking persons of Polish origin (BT1 and BT2), with medical and technical professions respectively, independently provided back-translations of the consensus version into English. Both translators were unfamiliar with the original questionnaire and its concept. During the meeting with all translators involved, all versions of the HOOS questionnaire were combined and a consensus on semantic, idiomatic, experiential and conceptual equivalence was reached, resulting in a pre-final version of the questionnaire.

#### Pilot-testing

The pre-final Polish version of the HOOS questionnaire was tested on 21 Polish native speaking outpatients with OA of the hip (9 men and 12 women with a mean and median age of 69 years, range 48–80 years). The patients completed the questionnaire in the presence of the project manager (PTP). Subjects were asked whether they fully understood the questions (items), whether they found any items ambiguous and whether they had any problems in answering them. The Polish version of the HOOS is available free of charge from www.koos.nu [5] (Supplementary material 1).

### Clinical validation study

The psychometric properties of the HOOS scale were evaluated according to the Consensus-based Standards for the selection of health Measurements Instruments (COSMIN) [7, 8].

### Patients

One hundred and eighty-three patients were eligible for THR at the Department of Orthopaedics, Ministry of the Interior and Administration Hospital in Olsztyn, Poland, over a three-year period between April 2013 and April 2016. All hip procedures were performed through the posterolateral approach. Patients had undergone either cementless (146 hips, 79%) or cemented (37 hips, 21%) THR.

**Fig. 1:**
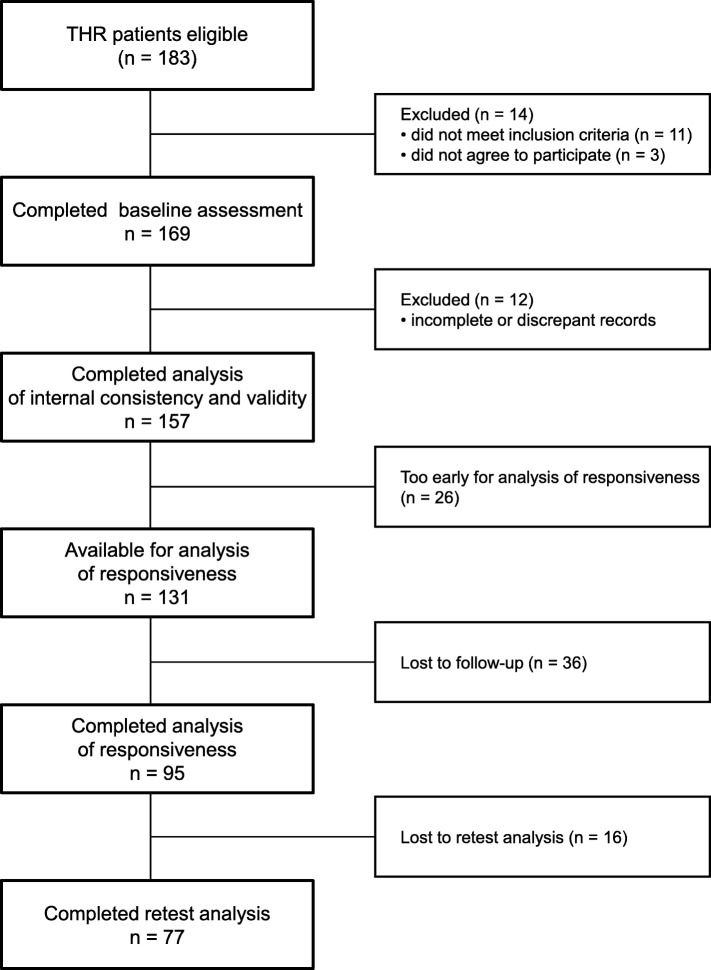
Flowchart presenting the study group formation

Inclusion criteria were: primary or secondary hip OA, according to the American College of Rheumatology criteria [9], ability to understand Polish written language and to understand and complete self-report questionnaires. Subjects with inflammatory arthritis, neurologic deficits, tumors and alcohol abuse were excluded from the study. Out of 183 subjects, 169 (92%) met the criteria and agreed to participate. Of those, 12 subjects were lost due to incomplete or discrepant records. Thus, 157 subjects formed the baseline study group (Fig. 1).

Data were collected three times: before THR (at baseline, for assessing internal consistency and validity), at routine follow-up 1 year after THR (for testing responsiveness) and, finally, one to 3 weeks after follow-up (for test-retest reliability).

The preoperative (baseline) and follow-up assessments were done in the clinic. During the preoperative assessment, the participants were asked to complete the Polish version of HOOS, the SF-36 and the Visual Analog Scale (VAS) for pain. At the follow-up assessment, the participants completed the HOOS questionnaire and the Global Perceived Effect (GPE) scale. For retest purposes, the HOOS questionnaire was completed once again, at home and returned by mail. All self-reported questionnaires, demographics and relevant information were processed by one orthopaedic surgeon (MKG).

### Questionnaires

#### HOOS

The HOOS is a 40-item self-administered hip-specific questionnaire including five subscales: Pain (10 items), Symptoms (5 items), Function in Daily Living (or Activity in Daily Living, ADL Function, 17 items), Sports and Recreation Function (4 items) and hip-related Quality of Life (QOL, 4 items). Each item is responded to by marking one of five response options from 0 (best) to 4 (worst) on a Likert scale. A normalized score from 0 (extreme problems) to 100 (no problems at all) are calculated separately for each subscale.

The user’s guide can be downloaded from www.koos.nu [5]. The format is user-friendly and the questionnaire takes about 10 min to complete. It is self-explanatory and patients can complete it in the waiting room or it can be used as a mailed survey.

#### SF-36

The SF-36 Health Survey is a generic self-administered questionnaire that includes 36 items, combined in eight health domains of which four cover physical health perceptions (Physical Functioning – PF, Role limitations because of physical problems – RP, Bodily Pain – BP, General Health – GH) and four mental health concepts (Vitality – VT, Social Functioning – SF, Role limitations because of emotional problems – RE and Mental Health – MH) [10]. A score from 0 (worst possible health status) to 100 (best possible health status) is independently generated for each domain as well as for two summary scores that have been extracted from the eight original scales and referred to as the Physical Component Summary (PCS) and Mental Component Summary (MCS). In order to prevent the inflation of the MCS scores by poor physical health scores that is observed when commonly used orthogonal-factor analytic model is used [11], scoring coefficients were calculated according to the oblique-factor analytic model [12].

SF-36 outcomes were calculated with Scoring Software v. 4.5 delivered by the copyrights holder (Optum Insight, Eden Prairie, MN, USA, license number QM018125). The SF-36 has already been validated in Polish [13].

#### VAS for pain

The VAS for pain is a simple way of measuring the intensity of pain. The 100-mm VAS is a unidimensional scale and it is considered valid and reliable [14].

#### GPE scale

The GPE scale is designed to quantify a patient’s improvement or deterioration over time, usually either to determine the effect of an intervention or to chart the clinical course of a condition [15, 16]. Patients were asked to rate their perceived hip condition after THR, at one-year follow-up, compared with the condition preoperatively. Patients had the following answer options: much better (3), better (2), somewhat better (1), no change (0), somewhat worse (− 1), worse (− 2) and much worse (− 3).

### Missing items

According to the 2013 Users’ Guide for the HOOS questionnaire, at least 50% of the items should be responded to. In our study, any missing data were handled according to the HOOS scoring instructions (available free of charge from www.koos.nu) with the participate mean substitution method. The missing data were imputed with the mean of the other values within the same subscale [5]. In addition, the multiple imputation method was used to verify the results. With this approach, any missing data from incomplete data sets were imputed to produce three complete data sets. Statistical analysis was then performed on each imputed data set and Cronbach’s α results were computed. Finally, the results were pooled to obtain a single Cronbach’s α [17].

SF-36 results were calculated using standard scoring procedures whereby missing values were replaced by scale means where valid responses were available for at least half of the scale items [10].

### Floor/ceiling effects

Floor or ceiling effects were determined preoperatively in patients that attended the baseline assessment, and 1 year after TKR in patients who were controlled at follow-up. They were considered to be present if more than 15% of the participants achieved either the lowest or the highest possible scores [18]. Comparisons of proportions for men and women with the lowest and the highest possible scores were evaluated with the McNemar’s test.

### Reliability

Reliability is an estimation of the consistency and stability of a measure. It includes an analysis of the extent to which a measure is internally consistent and free of measurement error [7].

#### Internal consistency

Internal consistency refers to an agreement between items on the same subscale and measures their degree of homogeneity. The internal consistency was assessed using Cronbach’s alpha coefficient [19] with 95% Feld’s confidential intervals (95% CI) and Pearson’s item to total (item-rest) correlation. Cronbach’s α was determined preoperatively and at follow-up. Cronbach’s α value of more than 0.70 was considered satisfactory [20]. An item-rest correlation greater than 0.50 was considered strong, between 0.35 and 0.5 moderate, and less than 0.35 weak [21].

#### Test-retest reliability

Test-retest reliability is the extent to which results of the same patient in the same health condition remain unchanged over time [8]. Test–retest reliability of the HOOS subscales was assessed at follow-up, twice, with one to 3 weeks interval. For test-retest studies, the time interval needs to be sufficiently short to ensure that no significant clinical change in the hip joint occurs and long enough to ensure that patients do not remember how they responded in the first questionnaire [22]. A retest interval between two days and 3 weeks is considered appropriate and has previously been used for the validation of the HOOS [23, 24].

Test–retest reliability of the HOOS was established by calculating the intraclass correlation coefficients (ICCs) (single measure, model 3, 1, two-way mixed model for absolute agreement) and 95% CI. ICCs between 0.75 and 0.90 were considered good and ICCs greater than 0.90 excellent [21, 25].

#### Measurement error

The measurement error is the systematic and random error of a patient’s score that is not attributed to true changes in the construct to be measured. The standard error of measurement (SEM) for absolute agreement of the test–retest reliability estimates how repeated measures of a person on the same instrument tend to be distributed around his or her ‘true’ score. SEM was calculated using one-way analysis of variance (ANOVA) square root of the within groups mean square value [26–28]. Then, in turn, the minimal detectable change (MDC), i.e. the smallest threshold of score change that is detectable and greater than random measurement error, was calculated using the formula: MDC=SEM × 1.96 × √2, where 1.96 derives from the 0.95% CI of no change and √2 represents two measurements evaluating the change [26, 29]. The MDC can be modified for group comparison, depending on the size of the group (*n* = 77), as follows: MDCgroup = MDCindividual/√n [30].

### Validity

#### Content validity

Content validity is assessed by making a judgment of relevance and comprehensiveness of the items. All subjects recruited for the study group were asked to assess whether the questionnaire items were relevant to their case and/or condition, whether the description of the construct was clear, and whether explanation of the domains was understandable.

#### Construct validity (hypotheses testing)

Construct validity is defined as the degree to which an instrument measures the characteristic to be measured. Basing on the assumptions of Terwee et al. [21], we examined the convergent construct validity of the Polish version of HOOS by testing an a priori set of hypotheses about the expected relationships between the HOOS subscales, the generic SF-36 measure and the VAS for pain at baseline.

In order to evaluate the association between domains, the Spearman’s rank correlation was used. Correlation coefficients greater than 0.5 were considered strong, correlations between 0.35 and 0.5 moderate, and less than 0.35 weak [31].

We expected the highest correlations when comparing the subscales that measure similar constructs. We hypothesized that:

1. since the HOOS subscale Pain and SF–36 BP measure a sufficiently similar construct, the correlation between these two measures should be strong and in the same direction,
2. the correlation between the HOOS subscale ADL Function and SF–36 PF should be moderate or strong and in the same direction,
3. the correlation between the HOOS subscale Sports and Recreation Function and SF–36 PF should be at least moderate and in the same direction,
4. the correlation between the HOOS subscale ADL Function and SF–36 PF should be higher than the correlation between the HOOS subscale ADL Function and the other subscales of the SF-36,
5. the correlation between the HOOS subscale Sports and Recreation Function and SF–36 PF should be higher than the correlation between the HOOS subscale Sports and Recreation Function and the other subscales of the SF-36,
6. the correlation between all HOOS subscales and PCS of the SF–36 should be strong and in the same direction,
7. all HOOS subscales should correlate stronger with PCS than with MCS of the SF–36,
8. the correlation between the HOOS subscale Pain and the VAS for pain should be moderate or strong and in the opposite direction.

### Responsiveness

Responsiveness is an ability of a measure to detect meaningful clinical change over time in the construct to be measured. It is critical for the use and application of a measure. We have expected to be able to detect clinical change that occurred following THR. As suggested by the COSMIN initiative, responsiveness was investigated formulating a priori hypotheses regarding expected 1) correlations of the HOOS score change with the GPE score and 2) effect sizes.

Associations between score change in the HOOS subscales and GPE were calculated with use of the Spearman’s rank correlation. Correlation coefficients greater than 0.5 were considered strong, correlations between 0.35 and 0.5 moderate, and less than 0.35 weak [32].

The standardized effect size (SES) was calculated in all HOOS subscales. It was defined as a mean score change divided by baseline SD (Kazis’ effect size) [33]. In addition to SES, responsiveness was also presented as standardized response mean (SRM). SRM was calculated by dividing the mean score change by the standard deviation of that score change [34]. Two hypotheses were formulated (a priori hypotheses 9 and 10):

9) the change in scores in all HOOS subscales between the baseline examination and follow-up control would correlate with the GPE score and that the correlation would be at least moderate.
10) SRM and ES should be higher for patients who reported their condition to be much better than in patients reporting to be better, somewhat better, no change, somewhat worse, worse and much worse in the GPE score.

### Statistical analysis

Descriptive statistics were used to describe sociodemographic and clinical characteristics preoperatively, at baseline and clinical characteristics after treatment, at follow-up. Data were checked for normality of distribution using the Kolmogorov–Smirnov test and tests for skewness and kurtosis. Since the data were normally distributed, the Student’s t-test was used to compare HOOS scores before THR and at follow-up.

Analyses were performed with the use of IBM SPSS Statistics for Windows V. 24.0.0 (IBM Corp. Armonk, New York, USA). We considered a two-tailed *p* value less than 0.05 to be significant.

## Results

### Linguistic and cross-cultural translation process

The translation process revealed some difficulties with the understanding of the description of activities that possibly cause pain (HOOS subscale Pain). Patients’ suggestions were reviewed and minor changes to the pre-final version were introduced. Clarifications of respective movements were added to item P2 “Straightening your hip fully” and to item P3” Bending your hip fully”. In addition, the expression “At night while in bed” in item P6 was supplemented with the phrase “Pain that bothers you while asleep”.

The revised version of the questionnaire was reassessed and found semantically, idiomatically and conceptually equivalent to the original version and then used in a clinical validation study. The Polish version of the pre-final HOOS questionnaire was well-accepted in the pre-test. All questions and response options were considered satisfactory and understandable by the subjects.

### Clinical validation study

#### Patients

Internal consistency and validity was studied in 157 patients (84 women, 73 men, aged 25–87 years) who participated in the preoperative (baseline) analysis. Follow-up was carried out between May 2014 and November 2016. Since the study was still ongoing in November 2016, 26 subjects had not completed a one-year period after the surgery and thus could not be analyzed for responsiveness. Out of 131 subjects eligible for follow-up, 36 dropped out (27%). Finally, the responsiveness analysis was performed in 95 patients (59 women, 36 men, aged 40–84 years) at a mean 1.1 years (0.9–1.9) after THR. Of these patients, 77 (46 women and 31 men, aged 43–84 years) completed the HOOS questionnaire twice for the test–retest reliability (response rate of 81%).

The median number of days from test to retest was 9 (ranging from 6 to 20).

To assess a possible inclusion bias, all patients from the baseline study group were analyzed with regard to age and gender, as well as their outcome in the HOOS subscales, SF-36 domains and VAS Pain. We found no significant differences in these characteristics between the subjects included in later analyses and those who were lost to follow-up. Patient characteristics are given in Table 1.

**Table 1:** Characteristics of patients who completed analysis of internal consistency, responsiveness and those who were lost to follow-up

Characteristics	At baseline		
	Completed analysis of consistency	Lost to follow-up	Completed analysis of responsiveness
*N* (% women)	157 (54)	36 (44)	95 (62)
Age at THR, mean [SD], years	66.8 (11.1)	68.8 (14.7)	67.0 (9.7)
**HOOS subscales**			
Pain	31.0 (16.5)	31.0 (17.7)	29.7 (16.1)
Symptoms	28.8 (17.9)	29.9 (17.1)	26.3 (17.7)
ADL	26.6 (14.4)	26.8 (16.4)	24.7 (16.6)
Sports/Rec	14.3 (14.4)	13.5 (15.0)	12.7 (13.0)
QOL	18.1 (15.6)	17.5 (15.5)	17.0 (15.6)
**SF-36**			
PF	19.5 (17.4)	21.1 (18.1)	18.1 (17.9)
RP	25.1 (19.2)	21.4 (16.3)	25.1 (19.8)
BP	18.6 (17.0)	17.9 (16.3)	18.0 (17.3)
GH	47.4 (14.7)	44.4 (15.6)	48.9 (15.1)
VT	39.2 (17.7)	35.7 (14.7)	40.2 (19.0)
SF	42.0 (27.4)	42.5 (24.1)	42.6 (28.8)
RE	65.5 (30.4)	64.5 (30.6)	63.3 (31.8)
MH	51.7 (18.7)	51.3 (15.7)	52.5 (20.5)
PCS	27.8 (6.4)	27.2 (6.1)	27.8 (6.5)
MCS	38.9 (10.5)	38.2 (9.4)	39.0 (11.2)
**VAS**	6.7 (1.7)	6.7 (1.8)	6.8 (1.7)

Due to the ongoing character of the study, 26 subjects had not completed one-year period after THR at time of follow-up assessment. Thus only 131 out of 157 subjects from the baseline group were eligible for further investigations. Since 36 subjects dropped out, the analysis of responsiveness was performed in 95 patients

*Abbreviations*: *SD* standard deviation, *THR* total hip replacement, *ADL* activities of daily living, *Sports/Rec* sports and recreation function, *QOL* quality of life, *PF* physical functioning, *RP* role-physical, *BP* bodily pain, *GH* general health, *VT* vitality, *SF* social functioning, *RE* role-emotional, *MH* mental health, *PCS* physical component summary, *MCS* mental component summary, *VAS* visual analog scale for pain

### Missing items

For the HOOS scale preoperatively, a total of 82 items of the possible 40 (number of items) × 157 (number of patients) were missing (1.31%). At follow-up, 51 of the possible 40 × 95 items were missing (1.34%), while at retest analysis, 50 of the possible 40 × 77 items were missing (1.62%). For the SF-36, the number of missing items at baseline was 5 (0.01%) of the possible 36 (items) × 157 (number of patients).

### Floor/ceiling effects

Preoperatively, there were neither ceiling effects, nor any patients with best possible scores in any of the HOOS subscales. The floor effects (indicating worst possible status) were found prior to THR for the subscales Sports and Recreation Function (24%) and QOL (25%). The worst possible scores were reported by 3% of the patients for the HOOS subscale Pain, 7% for Symptoms, and 5% for ADL. At follow-up, there were no ceiling effects in any HOOS subscales. The best possible scores were reported by 12% of the patients for the subscale Pain and Sport and Recreation Function, 14% for the subscale Symptoms, 13% for the subscale ADL and 7% for the subscale QOL. As expected, at follow-up there were neither floor effects nor any patients with the worst possible scores. No differences in the number of patients having the worst or best possible scores related to gender were observed (data not shown).

### Reliability

#### Internal consistency

Cronbach’s α for the HOOS subscales ranged from 0.76 to 0.95 at baseline and 0.87 to 0.97 at follow-up, indicating a good homogeneity of all items in the subscales. (Table 2). An analysis of Pearson’s correlations between each item and the total score (item-to-total correlations) in each subscale showed that all correlation coefficients were strong, except for item Q1 (“How often are you aware of your hip problem?”) preoperatively that was moderate (r_p_ = 0.48) (Table 2). When missing data were handled with the multiple imputation method, Cronbach’s α values obtained after pooling the results from the three data sets were similar to those achieved with the participate mean substitution approach. Differences in Cronbach’s α values and their 95% CI obtained with these two methods did not exceed 0.01. Item-to-total correlations calculated for data handled with multiple imputation method were in some cases (item A16 in the ADL Function subscale, SP4 in the Sports and Recreation Function subscale and Q4 in the QOL subscale) lower than those achieved when the participate mean substitution method was applied. Differences were not higher than 0.06, which did not change the strength of correlation in any case.

**Table 2:** Internal consistency of the HOOS subscales (*n* = 157)

HOOS subscales (number of items)	Cronbach’s alpha coefficients (95%CI)			Pearson’s item to total correlation	
	At baseline	At follow-up		At baseline	At follow-up
Pain (10)	0.92 (0.89 to 0.94)	0.95 (0.93 to 0.96)		0.59 to 0.81	0.71–0.85
Symptoms (5)	0.78 (0.72 to 0.83)	0.87 (0.83 to 0.91)		0.50 to 0.63	0.52–0.78
Function ADL (17)	0.95 (0.94 to 0.96)	0.97 (0.96 to 0.98)		0.55–0.80	0.64–0.87
Function sport/recreation (4)	0.86 (0.82 to 0.89)	0.89 (0.85 to 0.93)		0.69–0.74	0.58–0.85
QOL (4)	0.76 (0.69 to 0.82)	0.87 (0.83 to 0.91)		0.48–0.69	0.63–0.81

internal consistency was assessed with use of Cronbach’s alpha coefficient and Pearson’s item to total (item-rest) correlations. Both assessments were made for all HOOS subscales prior to primary THR (at baseline, n = 157) and at one-year follow-up (*n* = 95)

*Abbreviations*: *CI* confidence interval, *THR* total hip replacement, *ADL* activities of daily living, *Sports/Rec* sports and recreation function, *QOL* quality of life

#### Test–retest reliability

The HOOS questionnaire was completed within mean 10.8 days (SD 3.9, range 6–20 days). The reliability of all HOOS subscales was good or excellent, with ICCs ranging from 0.82 to 0.96 and SEM values between 4.32 and 9.46 (Table 3).

**Table 3:** Mean scores at test and retest follow-up, test-retest reliability and MDC values (n = 77)

HOOS subscales (number of items)	Mean HOOS score (SD)		ICC (95% CI)	SEM	Minimal detectable change (95% CI) in individuals	Minimal detectable change (95% CI) in group
	First follow-up assessment	Second follow-up assessment				
THR, n = 77						
Pain (10)	79.2 (19.4)	78.6 (21.6)	0.82 (0.72–0.88)	9.46	26.2	3.0
Symptoms (5)	78.2 (19.5)	78.4 (18.7)	0.92 (0.88–0.95)	5.41	15.0	1.7
ADL (17)	75.6 (21.2)	75.8 (21.1)	0.96 (0.94–0.97)	4.32	12.0	1.4
Sport/recreation (4)	56.5 (29.8)	57.8 (29.8)	0.91 (0.87–0.94)	8.82	24.4	2.8
QOL (4)	64.4 (24.4)	64.5 (23.0)	0.91 (0.87–0.94)	7.00	19.4	2.2

mean scores in the HOOS subscales (0 to 100, worst to best scale) are given at test (first follow-up) and retest (second follow-up) assessment one to 2 weeks apart. Test-retest reliability is presented as ICC values. MDC of HOOS subscales is presented for individuals and groups one year after primary THR

*Abbreviations*: *MDC* minimal detectable change, *THR* total hip replacement, *ICC* intraclass correlation coefficient, *SEM* standard error of measurement, *CI* confidence interval, *ADL* activities of daily living, *QOL* quality of life

#### Minimal detectable change

At the individual level, the MDC was lowest (12.0) for the HOOS subscale ADL Function, and highest (26.2) for the HOOS subscale Pain. At the group level, MDC ranged from 1.4 to 3.0 (Table 3).

### Validity

#### Content validity

All HOOS items were estimated to be relevant. The description of the domains was assessed to be understandable and the construct appeared to be clearly described. Thus, the items were assessed to be comprehensive.

#### Hypothesis testing

Seven out of eight a priori established hypotheses were supported. We confirmed a strong correlation between the subscales that intended to measure similar constructs: HOOS Pain vs SF-36 BP (r_s_ = 0.70, 95%CI 0.59 to 0.81) and HOOS Sports and Recreation Function and SF–36 PF (r_s_ = 0.71, 95%CI 0.59 to 0.82) (hypothesis 1 and 3, respectively). Noteworthy, the correlation between HOOS ADL Function vs SF-36 PF was strong (r_s_ = 0.68, 95%CI 0.56 to 0.80), as expected (hypothesis 2), but lower to the correlation between HOOS ADL Function and SF-36 BP (r_s_ = 0.73, 95%CI 0.62 to 0.84). Thus, hypothesis 4 was not confirmed.

The correlation between the HOOS subscale Sports and Recreation Function and SF–36 PF was at least 0.14 higher than the correlation with the other subscales of the SF-36 (hypothesis 5). We confirmed also a strong correlation between all HOOS subscales and the PCS of the SF–36 (r_s_ between 0.62, 95%CI 0.49 to 0.74 in the HOOS subscale Symptoms and 0.70, 95%CI 0.59 to 0.82 in the HOOS subscale ADL) (hypothesis 6). In addition, correlations of HOOS subscales with PCS were stronger than those with MCS of the SF–36 (hypothesis 7). All correlations between the HOOS subscales and the VAS-pain were moderate (hypothesis 8) (Table 4).

**Table 4:** Construct validity, given as Spearman’s correlations of five HOOS subscales, eight SF-36 subscales, PCS and MCS as well as VAS Pain in subjects following primary THR (n = 157)

		HOOS subscales				
		Pain	Symptoms	ADL	Sports/Rec	QOL
**SF-36 subscales**	PF	0.66^a^	0.60^a^	0.68^a^	0.71^a^	0.65^a^
		(0.54 to 0.78)	(0.47 to 0.73)	(0.56 to 0.80)	(0.59 to 0.82)	(0.53 to 0.77)
	RP	0.54^a^	0.49	0.55^a^	0.51^a^	0.54^a^
		(0.41 to 0.68)	(0.36 to 0.63)	(0.42 to 0.69)	(0.38 to 0.65)	(0.41 to 0.68)
	BP	0.70^a^	0.62^a^	0.73^a^	0.57^a^	0.61^a^
		(0.59 to 0.81)	(0.49 to 0.74)	(0.62 to 0.84)	(0.44 to 0.70)	(0.49 to 0.74)
	GH	0.17	0.23	0.20	0.16	0.01
		(0.01 to 0.32)	(0.08 to 0.39)	(0.04 to 0.35)	(0.01 to 0.32)	(−0.15 to 0.17)
	**VT**	**0.37**	**0.46**	**0.38**	**0.36**	**0.30**
		**(0.22 to 0.52)**	**(0.32 to 0.60)**	**(0.23 to 0.53)**	**(0.21 to 0.51)**	**(0.15 to 0.45)**
	**SF**	**0.30**	**0.47**	**0.34**	**0.29**	**0.34**
		**(0.15 to 0.46)**	**(0.33 to 0.61)**	**(0.20 to 0.50)**	**(0.14 to 0.45)**	**(0.19 to 0.49)**
	**RE**	**0.11**	**0.29**	**0.10**	**−0.08**	**0**
		**(−0.04 to 0.27)**	**(0.13 to 0.44)**	**(−0.06 to 0.26)**	**(−0.24 to 0.08)**	**(− 0.16 to 0.16)**
	**MH**	**0.32**	**0.38**	**0.33**	**0.20**	**0.23**
		**(0.17 to 0.47)**	**(0.23 to 0.52)**	**(0.18 to 0.48)**	**(0.05 to 0.36)**	**(0.07 to 0.38)**
	PCS	0.66^a^	0.62^a^	0.70^a^	0.69^a^	0.64^a^
		(0.54 to 0.78)	(0.49 to 0.74)	(0.59 to 0.82)	(0.57 to 0.81)	(0.51 to 0.76)
	**MCS**	**0.28**	**0.45**	**0.29**	**0.13**	**0.18**
		**(0.13 to 0.43)**	**(0.31 to 0.59)**	**(0.14 to 0.44)**	**(−0.02 to 0.29)**	**(0.02 to 0.33)**
**VAS**	Pain	−0.49	−0.42	− 0.47	−0.40	− 0.37
		(−0.62 to − 0.34)	(− 0.56 to − 0.27)	(−0.61 to − 0.33)	(−0.55 to − 0.25)	(−0.52 to − 0.22)

As hypothesized, correlations between HOOS subscales and the PCS of the SF-36 were stronger than between HOOS subscales and the MCS of the SF-36 (a priori hypothesis 7). Note that the subscales better representing Mental Health as well as MCS scores appear in the table in bold font

*Abbreviations*: *THR* total hip replacement, *ADL* activities of daily living, *Sports/Rec* sports and recreation function, *QOL* quality of life, *PF* physical functioning, *RP* role-physical, *BP* bodily pain, *GH* general health, *VT* vitality, *SF* social functioning, *RE* role-emotional, *MH* Mental Health, *PCS* physical component summary, *MCS* mental component summary, *VAS* visual analog scale for pain

^a^ As hypothesized, expected correlations were above 0.50 for a priori hypotheses 1–3 and 6

### Responsiveness

The HOOS scores from all subscales increased significantly (*p* <  0.001) at one-year follow-up after THR as compared to preoperative values (Table 5). All patients examined reported improvement in their hip condition at follow-up scoring ‘somewhat better’, ‘better’, or ‘much better’ in the GPE score (GPE ranging 1–3). There were no subjects who scored ‘no change’, ‘somewhat worse’, ‘worse’ or ‘much worse’. A moderate correlation was observed between GPE score and score change in the HOOS subscales: Sports and Recreation Function and HOOS and QOL (r_s_ = 0.38, 95%CI 0.19 to 0.57, and r_s_ = 0.43, 95%CI 0.25 to 0.62 respectively). In all other subscales, correlations were weak (r_s_ ranging 0.27–0.32) (Table 5). The a priori hypothesis 9 could thus be supported partially. The responsiveness measured with the SES and SRM for entire group was high for all subscales, with SES ranging from 2.91 in the subscale Symptoms to 3.58 in the Sports and Recreation Function and SRM ranging from 1.73 in the subscale Sports and Recreation Function to 2.43 in the subscale ADL Function (data not shown). Since patients who described their hip condition at follow-up as ‘much better’ reported higher responsiveness (in both SES and SRM) in all five HOOS subscales than those who scored ‘better’ or ‘somewhat better’ (Table 5), the a priori hypothesis 10 could be confirmed.

**Table 5:** Mean scores (at baseline and at follow-up) and responsiveness of the HOOS subscales (n = 95)

HOOS subscales	Mean score (SD)		***P***	GPE score	“Much better”, n = 77		Others, ***n*** = 18	
	At baseline	At follow-up		Spearman r (95% CI)	SES	SRM	SES	SRM
Pain	29.7 (16.1)	79.2 (18.5)	< 0.001	0.28 (0.08–0.48)	3.29	2.74	2.17	1.69
Symptoms	26.3 (17.7)	77.8 (18.9)	< 0.001	0.32 (0.12–0.51)	3.13	2.60	1.97	1.67
ADL	24.7 (16.6)	75.9 (20.6)	< 0.001	0.27 (0.07–0.47)	3.33	2.71	2.18	1.75
Sports/Rec	12.7 (13.0)	59.2 (28.8)	< 0.001	0.38* (0.19–0.57)	3.93	1.90	1.97	1.81
QOL	17.0 (15.6)	64.8 (22.9)	< 0.001	0.43* (0.25–0.62)	3.44	2.34	1.50	0.92

mean HOOS scores (0 to 100, worst to best scale) are given in subjects prior to primary THR and at one-year follow-up (*n* = 95). Responsiveness is given as Spearman’s correlations of the five HOOS subscales and GPE score. Standardized effect size (SES) and standardized response mean (SRM) was calculated separately in patients who scored ‘much better’ (*n* = 77) and in other participants (those who scored ‘somewhat better’ and ‘better’) (*n* = 18). There were no subjects who scored ‘no change’, ‘somewhat worse’, ‘worse’ or ‘much worse’

*Abbreviations*: *THR* total hip replacement, *GPE* global perceived effect, *ADL* activities of daily living, *QOL* quality of life, *SES* standardized effect size, *SRM* standardized response mean

* Correlations above 0.35 for a priori hypothesis 9

## Discussion

Our study reports on the linguistic and cross-cultural translation and the psychometric properties of the Polish version of the HOOS in patients after THR. The study was performed in accordance with the COSMIN guidelines recommended for validation processes [8, 35].

The Polish version of the HOOS questionnaire was easy to fill in and understandable for patients; they did not need any supplementary instructions to answer the questions independently. This resulted in a high percentage of answers and a low percentage of missing data.

A systematic literature search for psychometric assessment of OA questionnaires allowed Veenhof et al. [36] to conclude that the HOOS questionnaire was one of the top three measures with the best ratings for its psychometric properties to assess both pain and physical function. Since then, the HOOS has been extensively studied and validated in several languages [23, 37–43]. All these studies have confirmed that the HOOS questionnaire was reliable, valid and responsive to patient perceptions of hip problems.

In the present study, we found floor effects preoperatively for the HOOS subscales Sports and Recreation Function and QOL. This observation could have been expected since these two subscales were developed as an extension of the WOMAC for younger, more active subjects, thus appeared to be more sensitive and discriminative for older and disabled patients with OA than original WOMAC subscales [2].

The Polish version of the HOOS questionnaire has a good internal consistency both preoperatively and at follow-up. Since the Cronbach’s alpha values were markedly higher than 0.7 preoperatively and even 0.8 at follow-up, all subscales of the HOOS questionnaire could be considered reliable. Internal consistency for the subscales Symptoms, Sports and Recreation Function and QOL were, however, slightly lower than that observed in respective subscales in a previous study evaluating psychometric properties of the Polish version of the KOOS questionnaire in patients with OA undergoing total knee replacement [44]. A lower value of alpha could be due to a heterogeneous construct of these subscales. Cronbach’s alpha was greatest for the ADL subscale both preoperatively and at follow-up (0.95 and 0.97 respectively), which concurs with previous validation studies (0.94 in the French version, and 0.98/0.95 for OA/THR group in the Dutch version [23], 0.96 in the Korean [38], German [37] and Italian [45] versions and 0.97 in the Japanese version [42]. However, since it had been reported [46, 47] that subscales showing a high coefficient alpha are not necessarily homogenous or unidimensional, very high Cronbach’s alpha (exceeding 0.9) may suggest that some items of both, the 17-item ADL Function subscale, and the 10-item Pain subscale are redundant as they test the same question in a different guise. Indeed, exploratory principal factor analysis confirmed item redundancy in both subscales (Supplementary material 2).

It has been known, however, that removal of redundant items cannot only make the measurement instrument more reliable but also can easily affect both the content and construct [46, 47]. Since it was not our purpose to develop a new instrument or to revise the existing one, we did not change the questionnaire structure and extract any items from the subscales. Consequently, we accepted that Polish version of the HOOS was multidimensional and that it contained some items that loaded on more than one factor.

Findings from internal consistency analysis in terms of Pearson item-total correlations suggested that all items of the HOOS questionnaire were correlated among themselves within the subscales.

We have found that test-retest for all HOOS subscales was good or excellent, with ICCs ranging from 0.82 to 0.96. This observation is in accordance with previous validation studies [3, 23, 37, 39, 41] and proves that the Polish version of the HOOS was stable and reproducible in the patients examined. In the present study, the highest ICCs were observed in the HOOS subscale ADL Function. The possible explanation is that the questions about daily activities in stable patients after THR were less discriminative than in other subscales.

Another important finding in this study was that the changes observed in all HOOS subscales were clinically and statistically meaningful at the group level. The MDC value for groups was found to be between 1.4 and 3.0 points in different subscales, which indicates that the Polish version of the HOOS has an ability to detect differences of more than 3 points between the measurements. As expected, the sensitivity of the HOOS subscales was lower at the individual level. The MDC should preferably be smaller than the other important benchmark, not calculated here, minimal important change (MIC) that is the smallest change score needed for the effect to be considered clinically relevant. A MIC of 8–10 points was considered to be appropriate for different KOOS subscales [2] and seems to be convenient even for HOOS subscales. The MDC value of 12 points that was detected in the HOOS subscale ADL was at slightly higher level as the MIC, however not small enough to be classified as clinically relevant even for individual subjects. Since MDCs for other subscales should be between 15 and 26 to be considered remarkable with 95% confidence, they could not be easily detected in individuals. MDC values obtained in our study were higher than those observed by Ornetti et al. [41] and similar to MDCs reported by Naylor et al. [40] which ranged from 18 to 24. The smallest MIC values, between 6.1 and 8.6 score points, have been presented by Arbab et al. [37] who validated the German version of the HOOS. These low values might be related to large size of the study group (251 patients) and to the fact that MDC was calculated basing on the confidence level of 90%.

Since there are no other instruments evaluating pain and function related to the hip validated in Polish, construct validity of the HOOS was determined only by comparing the HOOS subscales with the subscales of the generic measure SF-36. As expected, we found strong correlations between subscales of the HOOS and SF-36 that were intended to measure similar constructs. The correlation values were comparable to those reported by de Groot et al. [23], Satoh et al. [42] and Torre et al. [45] in THR patients with a mean age of 62–66 years and higher than observed by Nilsdotter et al. in Swedish patients over 70 years of age [4]. This observation might have been expected since the outcome in THR is not specific to the joint but to overall impact on health, and therefore sensitive to age.

In analysis of the construct validity we confirmed all a priori hypotheses except for hypothesis 4 in which we expected that the HOOS subscale ADL Function correlates better with SF–36 PF than other SF-36 subscales. Unexpectedly, we observed that the correlation between HOOS ADL Function and SF-36 BP was even stronger than between ADL and PF. This observation may obviously give some difficulty in interpreting the results.

The choice of the responsiveness parameter depends on the focus of interest and the characteristics of the different methods, as outlined in the background. In this study, the HOOS ability to detect clinically relevant changes over time was assessed with use of the GPE. A correlation of at least 0.35 was observed between the GPE score and the score change in HOOS subscales Sports and Recreation Function and QOL. We expected, however, that such effective intervention as THR would be more responsive even within other domains. In our study, all patients reported their hip conditions to be at least ‘better’ than prior to operation. Correlations between the GPE and HOOS score changes would certainly be much higher if patients who did not improve or worsened in the HOOS score reported no change or even deterioration of their hip condition over time. Furthermore, we have found that even confidence intervals of correlation between GPE and HOOS subscales were much wider than those computed in the assessment of correlation between the results of HOOS subscales and SF-36 domains. In our opinion, this may eventuate from the distribution of variables rather than the sample size.

The follow-up questionnaires were completed during hospital visit and gathered by the same surgeon who earlier performed the THR surgeries. Since the GPE questions are put more directly than the items in the HOOS subscales, patients who answer them feel more comfortable when they elevate the score.

We observed large values of SES and SRM. This result may have been expected since THR is the most effective hip intervention. Our results were superior to those reported in other HOOS validation studies with median follow-up of 3 to 7 months [4, 41, 42]. Patients in the present study were assessed for responsiveness at a mean 1.1 years after THR, a period that is thought to be sufficient for adaptation to the new health status [4]. In summary, the results of the responsiveness assessment confirmed that both, the HOOS are able to recognize clinical improvement in patients undergoing THR.

The study’s strength is that we examined a well-defined, relatively large and likely to be representative group of patients with end-stage hip OA undergoing THR. The age and gender profiles of the study participants reflect those of the entire patient populations undergoing THR, as reported in international registries [48, 49]. A single-group design is, however, also a limitation of this study. The subjects assessed did not represent the entire spectrum of patients with hip OA. Elderly patients with end-stage OA have more pain and are not able to maintain a high level of physical activity and are thus are limited in their everyday life more than younger subjects with early OA. Further investigation concerning the psychometric properties in younger patients with hip dysfunctions and earlier stages of OA is advised.

The rate of loss to follow-up in the presented study was approximately 27%. However, since patients who were lost to follow-up and those who were eligible for analysis of responsiveness and test-retest reliability had similar baseline results in all HOOS and SF-36 subscales we believe that this is not a serious limitation.

Although there is no gold standard of construct validity assessment, the fact that construct validity was analysed only by assessing the relationship between the HOOS subscales with matching domains of the SF-36 can be regarded as another weakness of the study. However, up to date, there are no instruments evaluating hip-related pain and function validated in Polish that could be compared with the HOOS and used in the assessment of construct validity.

## Conclusions

The Polish version of HOOS demonstrated good psychometric properties and appears to be useful for the evaluation of patient-relevant outcome in subjects with hip OA undergoing THR. Since MDCs for the HOOS subscales, are substantially higher than MIC and thus cannot be detected at an individual level, the Polish version of the HOOS is advocated for assessment of groups of patients.

## Supplementary information

**Additional file 1.** Polish-adapted Hip disability and Osteoarthritis Outcome Score (HOOS).

**Additional file 2.** Factor analysis of the Polish version of the HOOS.

## Data Availability

The datasets generated and analyzed during the current study are available from the corresponding author on reasonable request.

## Abbreviations

ADL: Activities of daily living
BP: Bodily pain
CI: Confidence interval
COSMIN: Consensus-based Standards for the selection of health Measurements Instruments
ES: Effect size
GPE: Global perceived effect
HOOS: Hip disability and osteoarthritis outcome score
ICC: Intraclass correlation coefficient
KMO: Kaiser-Meyer-Olkin measure
KOOS: Knee injury and osteoarthritis outcome score
MDC: Minimal detection change
MIC: Minimal important change
OA: Osteoarthritis
PF: Physical functioning
PRO: Patient-reported outcome
QOL: Quality of life
RP: Role-physical
GH: General health
MH: Mental health
RE: Role-emotional
SD: Standard deviation
SEM: Standard error of measurement
SES: Standardized effect size
SF: Social functioning
SF-36: Short Form 36
SRM: Standardized response mean
THR: Total hip replacement
VAS: Visual Analogue Scale
VT: Vitality
WOMAC: Western Ontario and McMaster Universities Osteoarthritis Index
