# Supplementary material for: Polish adaptation and validation of the hip disability and osteoarthritis outcome score (HOOS) in osteoarthritis patients undergoing total hip replacement
Source: Health Qual Life Outcomes. 2020 May 12;18:135. doi: 10.1186/s12955-020-01390-4 (PMC7216355; doi:10.1186/s12955-020-01390-4)
Supplement: Supplementary file 1 — Additional file 1. Polish-adapted Hip disability and Osteoarthritis Outcome Score (HOOS). [file 12955_2020_1390_MOESM1_ESM.pdf]

HOOS
ANKIETA DLA CHORYCH Z DOLEGLIWOŚCIAMI STAWU BIODROWEGO

Dzisiejsza data…………………………………………………………………….

Data urodzenia…………………………………………………………………….

Imię i nazwisko…………………………………………………………………….

INSTRUKCJA: Niniejszy kwestionariusz dotyczy Twojej własnej oceny stanu biodra. Informacja ta pomoże nam zrozumieć, jakim problemem jest dla Ciebie chore biodro i jak sprawnie możesz podołać czynnościom dnia codziennego.
Odpowiedz na każde pytanie, stawiając krzyżyk w odpowiedniej **i tylko jednej** kratce dla każdego pytania. Jeśli nie jesteś pewny/pewna jak odpowiedzieć, zaznacz tę możliwość, która wyda Ci się najbliższa prawdy.

Objawy
Odpowiedz na poniższe pytania, biorąc pod uwagę objawy, które wystąpiły w czasie **ostatniego tygodnia**.

S1 Czy czujesz chrzęszczenie/tarcie, słyszysz ”strzelanie” lub jakikolwiek inny dźwięk w stawie biodrowym?

nigdy	rzadko	czasami	często	zawsze
☐	☐	☐	☐	☐

S2 Czy masz trudności, kiedy chcesz stanąć w szerokim rozkroku?

żadnych	lekkie	średnie	duże	bardzo duże
☐	☐	☐	☐	☐

S3 Czy masz trudności, kiedy podczas marszu chcesz wydłużyć krok?

żadnych	lekkie	średnie	duże	bardzo duże
☐	☐	☐	☐	☐

Sztywność
Poniższe pytania dotyczą stopnia sztywności stawu biodrowego, którą zaobserwowałeś w ciągu **ostatniego tygodnia**. Sztywność jest uczuciem ograniczenia lub zmniejszenia swobody ruchu w stawie biodrowym.

S4 Jak duża jest sztywność Twojego biodra tuż po pierwszym wstaniu z łóżka?

nie ma	łagodna	średnia	duża	bardzo duża
☐	☐	☐	☐	☐

S5 Jak duża jest sztywność Twojego biodra po siedzeniu, leżeniu bądź odpoczynku **w ciągu dnia**?

nie ma	łagodna	średnia	duża	bardzo duża
☐	☐	☐	☐	☐

Ból

P1 Jak często odczuwasz ból biodra?

nigdy	raz w miesiącu	raz w tygodniu	każdego dnia	cały czas
☐	☐	☐	☐	☐

Jak silny ból stawu biodrowego odczuwałeś/-aś w ciągu **ostatniego tygodnia** podczas wykonywania poniższych czynności?

P2 pełny wyprost biodra (uniesienie nogi do góry w leżeniu na brzuchu)

żadnego	lekki	średni	duży	nie do wytrzymania
☐	☐	☐	☐	☐

P3 pełne zgięcie biodra (podciągnięcie kolan do klatki piersiowej w leżeniu na plecach)

żadnego	lekki	średni	duży	nie do wytrzymania
☐	☐	☐	☐	☐

P4 chodzenie po płaskim podłożu

żadnego	lekki	średni	duży	nie do wytrzymania
☐	☐	☐	☐	☐

P5 chodzenie po schodach

żadnego	lekki	średni	duży	nie do wytrzymania
☐	☐	☐	☐	☐

P6 leżenie w łóżku w nocy (ból, który przeszkadza we śnie)

żadnego	lekki	średni	duży	nie do wytrzymania
☐	☐	☐	☐	☐

P7 siedzenie lub leżenie

żadnego	lekki	średni	duży	nie do wytrzymania
☐	☐	☐	☐	☐

P8 stanie w pozycji wyprostowanej

żadnego	lekki	średni	duży	nie do wytrzymania
☐	☐	☐	☐	☐

P9 chodzenie po twardym podłożu (np. asfalt, beton)

żadnego	lekki	średni	duży	nie do wytrzymania
☐	☐	☐	☐	☐

P10 chodzenie po nierównym podłożu

żadnego	lekki	średni	duży	nie do wytrzymania
☐	☐	☐	☐	☐

Czynności życia codziennego

Następne pytania dotyczą normalnej aktywności fizycznej. Chcemy ocenić Twoją zdolność do przemieszczania się i wykonywania codziennych czynności. Dla każdej wymienionej czynności określ stopień trudności, jaki napotykasz przy jej wykonywaniu z uwagi na Twoje biodro.

A1 schodzenie ze schodów

żaden	lekki	średni	duży	bardzo duży
☐	☐	☐	☐	☐

A2 wchodzenie po schodach

żaden	lekki	średni	duży	bardzo duży
☐	☐	☐	☐	☐

A3 wstanie z pozycji siedzącej

żaden	lekki	średni	duży	bardzo duży
☐	☐	☐	☐	☐

Dla wymienionych niżej czynności wskaż stopień trudności, który odczuwałeś/-aś w związku ze swoim biodrem w ciągu **ostatniego tygodnia**.

A4 stanie

żaden	lekki	średni	duży	bardzo duży
☐	☐	☐	☐	☐

A5 skłon do podłogi/podniesienie przedmiotu z podłogi

żaden	lekki	średni	duży	bardzo duży
☐	☐	☐	☐	☐

A6 chodzenie po płaskim podłożu

żaden	lekki	średni	duży	bardzo duży
☐	☐	☐	☐	☐

A7 wsiadanie i wysiadanie z samochodu

żaden	lekki	średni	duży	bardzo duży
☐	☐	☐	☐	☐

A8 robienie zakupów

żaden	lekki	średni	duży	bardzo duży
☐	☐	☐	☐	☐

A9 wkładanie skarpet/pończoch/rajstop

żaden	lekki	średni	duży	bardzo duży
☐	☐	☐	☐	☐

A10 wstawanie z łóżka

żaden	lekki	średni	duży	bardzo duży
☐	☐	☐	☐	☐

A11 zdejmowanie skarpet/pończoch/rajstop

żaden	lekki	średni	duży	bardzo duży
☐	☐	☐	☐	☐

A12 leżenie w łóżku (z przekręcaniem się na boki i zachowaniem tej samej pozycji biodra przez dłuższy czas)

żaden	lekki	średni	duży	bardzo duży
☐	☐	☐	☐	☐

A13 wchodzenie i wychodzenie z wanny/spod prysznica

żaden	lekki	średni	duży	bardzo duży
☐	☐	☐	☐	☐

A14 siedzenie

żaden	lekki	średni	duży	bardzo duży
☐	☐	☐	☐	☐

A15 siadanie na sedesie, wstawanie z sedesu

żaden	lekki	średni	duży	bardzo duży
☐	☐	☐	☐	☐

Dla wymienionych niżej czynności wskaż stopień trudności, który odczuwałeś/-aś w związku ze swoim biodrem w ciągu **ostatniego tygodnia**.

A16 wykonywanie ciężkich prac domowych (przesuwanie ciężkich przedmiotów, mebli, szorowanie podłóg itp.)

żaden	lekki	średni	duży	bardzo duży
☐	☐	☐	☐	☐

A17 wykonywanie lekkich prac domowych (ścieranie kurzu, gotowanie itp.)

żaden	lekki	średni	duży	bardzo duży
☐	☐	☐	☐	☐

Aktywność sportowa i rekreacyjna

Pytania dotyczą czynności podczas wzmożonej aktywności fizycznej. Jaka była trudność wykonania wymienionych czynności ze względu na stan Twojego biodra w ciągu **ostatniego tygodnia**?

SP1 przysiady / kucanie

żadna	lekka	średnia	duża	nie do wykonania
☐	☐	☐	☐	☐

SP2 bieganie

żadna	lekka	średnia	duża	nie do wykonania
☐	☐	☐	☐	☐

SP3 kręcenie i obracanie się na pięcie/stopie

żadna	lekka	średnia	duża	nie do wykonania
☐	☐	☐	☐	☐

SP4 chodzenie po nierównym podłożu

żadna	lekka	średnia	duża	nie do wykonania
☐	☐	☐	☐	☐

Jakość życia

Q1 jak często biodro daje Ci znać o sobie?

nigdy	raz w miesiącu	raz w tygodniu	każdego dnia	stale
☐	☐	☐	☐	☐

Q2 czy zmieniłeś/-aś swój styl życia, aby unikać potencjalnie niebezpiecznych czynności dla swojego biodra?

w ogóle	trochę	średnio	bardzo	całkowicie
☐	☐	☐	☐	☐

Q3 w jakim stopniu możesz polegać na sprawności swojego biodra?

całkowicie	bardzo	średnio	trochę	w ogóle
☐	☐	☐	☐	☐

Q4 Ile ogólnie kłopotu sprawia Ci biodro?

w ogóle	trochę	średnio	dużo	bardzo dużo
☐	☐	☐	☐	☐

Dziękujemy bardzo za dokładne wypełnienie ankiety
