# Supplementary material for: Polish adaptation and validation of the hip disability and osteoarthritis outcome score (HOOS) in osteoarthritis patients undergoing total hip replacement
Source: Health Qual Life Outcomes. 2020 May 12;18:135. doi: 10.1186/s12955-020-01390-4 (PMC7216355; doi:10.1186/s12955-020-01390-4)
Supplement: Supplementary file 2 — Additional file 2. Factor analysis of the Polish version of the HOOS. [file 12955_2020_1390_MOESM2_ESM.docx]

**ADDITIONAL FILE 2.**

**TITLE:**

Polish adaptation and validation of the Hip disability and Osteoarthritis Outcome Score (HOOS) in osteoarthritis patients undergoing total hip replacement. Explanatory principal factor analysis.

**MATERIAL AND METHODS**

**Validity**

*Structural validity (exploratory principal factor analysis)*

The factor analysis is a method designed to determine if the observed variables (items) could be explained by a smaller number of latent variables (called factors). In order to identify factor structure of the Polish version of HOOS, an exploratory factor analysis was carried out. Investigations were conducted on all items of the HOOS scale with use of principal component analyses with the orthogonal rotation procedure (Varimax). The Kaiser-Meyer-Olkin (KMO) measure was used to determine whether the variables within the data set shared a common factor with other variables. KMO values greater than 0.8 were considered meritorious, those greater than 0.9 marvellous. According the Kaiser’s criterion [1], factors with eigenvalue greater than 1 were extracted. The scree plot of the correlation matrix of all items was drawn. Factors that appeared over the point where the curve bends (“elbow”) were considered to be meaningful [2]. An analysis of the factor structure and loading was made. Factor loading of 0.4 or above was defined as substantial loading and desirable for an item to be significant. The subscale item that had a substantial loading on more than one factor (cross-loading), was considered to be “complex”, meaning that it had an affinity to two or more of the derived factors and it did not describe the same aspect. The results are given as percentage of variance in the subscale score explained by the principal factor(s).

**RESULTS**

**Validity**

*Structural validity (exploratory principal factor analysis)*

The KMO measure of sampling adequacy was marvellous (0.94), which suggested that the sample was adequate for an exploratory factor analysis. The scree plot confirmed the retention of the first six factors. Thus, six factors were sufficient to describe the data. This solution accounted for 68% of the total variance for the Polish version of the HOOS questionnaire (with eigenvalues of 19.5, 2.2, 1.7, 1.4, 1.2 and 1.1 for respective factors).

In the HOOS subscale Pain, items P4, P5 and P8-P10 loaded substantially on the first factor (ranging between 0.53 and 0.78). The P1 item had a substantial loading on the sixth factor (0.66), item P3 on the fourth factor (0.57), while items P6 and P7 on the third factor (0.73 and 0.74 respectively). In the case of item P2, a cross-loading of both the first (0.44) and the fourth factor (0.55) was observed.

In the HOOS subscale symptoms, items S2 and S3 loaded substantially on the sixth factor (0.61 in both items), while items S4 and S5 loaded on the fifth factor (0.71 and 0.8 respectively).

Four out of 17 items from the subscale ADL Function (A1, A2, A4 and A6) had a substantial loading on only the first factor (ranging between 0.48 and 0.73). Items A7 and A16 loaded on only the second factor (0.49 and 0.63 respectively), items A12 and A14 loaded on only the third factor (0.74 in both items) and items A9 and A11 only the fourth factor (0.73 and 0.7, respectively). In all other items, the cross-loading of different combination of factors was observed. Item A3 loaded on the first and the fifth factor (0.53 and 0.45, respectively), A13 on the first and the fourth factor (0.47 and 0.46), and A17 on the first and the second factor (0.66 and 0.41). Items A5 and A15 loaded on the second factor (0.41 in both cases) in combination with the fourth (0.59) and the third (0.52), respectively. Item A10 (“rising from bed”) loaded on three factors: the first, the second and the third (0.45, 0.40 and 0.42, respectively).

Items SP1-SP3 from the subscale Sports and Recreation Function loaded highly on the second factor (ranging from 0.64 to 0.74). In the SP4 item, a cross-loading of the second and the first factor (0.46 and 0.51, respectively) was observed.

In the subscale Quality of Life, items QOL2-QOL4 loaded substantially on the second factor (ranging between 0.64 and 0.66) whereas item QOL1 loaded on only the sixth factor (0.42) (data not shown).

**DISCUSSION**

The current study is one of the first to test the factor structure of the HOOS in a specific language (after the original Swedish validation study [3]). With respect to the dimensional structure of the HOOS questionnaire, we found that the Polish version of the HOOS contains six principal factors. The factor analysis revealed that although the Cronbach’s alpha and Pearson item-total correlations of our dataset were high, the dataset was not unidimensional. In our study, a large first eigenvalue (19.5) and much smaller subsequent eigenvalues (2.2 and less) suggested that there was a leading global factor. Indeed, the first factor dominated in 14 items in the HOOS subscales Pain and ADL Function and in one item in the subscale Sports and Recreation. We observed that seven items loaded on more than one factor. One item did not load on any factor (S1: Do you feel grinding, hear clicking or any other type of noise when your hip moves?) and thus it could be questioned what aspect it measured.

It should not be surprising that the HOOS subscale Pain, the subscale Symptoms and the subscale ADL Function captured more than one principal factor and that the same factor (global factor) was loaded on through several subscales. Although this multidimensionality did not substantially contribute to reliability of the subscales, it certainly requires a cautious interpretation.

In the validation study of the original Swedish version of the HOOS, Nilsdotter et al. [3] carried out an item-selection procedure through factor analysis of 51 items in five subscales. The authors found that some of the items in each subscale loaded on more than one factor. For that reason, 11 items were not included in the final version of the HOOS questionnaire though they were relevant and had a sufficient responsiveness. Such procedures are recommended in order to maximize the internal consistency, as well as convergent and discriminant validity of multidimensional scale [4]. One possible approach to do it is to, initially, define preliminary scales through item-total correlations and/or exploratory factor analysis, then to examine the scale’s unidimensionality through a confirmatory factor analysis, and, finally, to assess the reliability of the scale through internal-consistency analyses [5]. Another opportunity consists in performing sequential approach that starts off with analysis of internal consistency followed by convergent and discriminant validity analyses [4]. In this stepwise procedure, a subscale’s reliability is optimised (often maximised) by removing the least reliable item or items, as indicated by the expected increase (if any) in Cronbach’s alpha for the subscale.

As has already been mentioned, Cronbach’s alpha values and Pearson item-total correlations were high in our study. However, since it had been reported [6, 7] that items showing a high coefficient alpha are not necessarily homogenous or unidimensional, very high Cronbach’s alpha (exceeding 0.95) may suggest that some items of the 17-item ADL Function subscale are redundant as they test the same question in a different guise. Indeed, when seven items that loaded on more than one factor, as calculated in the explanatory factor analysis, were removed from the subscale, Cronbach’s alpha decreased to 0.90 (95% CI 0.88-0.93). Similarly, it seems that even the 10-item Pain subscale might present some item redundancy, as confirmed by the follow-up Cronbach’s alpha value of 0.95. We found that the nine-item version of the subscale might be sufficient to cover the construct since this lowers the alpha value to 0.91 (0.88-0.93).

It has been known, however, that removal of redundant items cannot only make the measurement instrument more reliable but also can easily affect both the content and construct [8]. Since it was not our purpose to develop a new instrument or to revise the existing one, we did not change the questionnaire structure and extract any items from the subscales. Consequently, we accepted that Polish version of the HOOS was multidimensional and that it contained some items that loaded on more than one factor.*

**REFERENCES**

1. Kaiser HF: **The application of electronic computers to factor analysis. .** *Educ Psychol Meas* 1960, **20:**141-151.

2. Cattell RB: **The Scree Test For The Number Of Factors.** *Multivariate Behav Res* 1966, **1:**245-276.

3. Nilsdotter AK, Lohmander LS, Klässbo M, Roos EM: **Hip disability and osteoarthritis outcome score (HOOS)--validity and responsiveness in total hip replacement.** *BMC Musculoskelet Disord* 2003, **4:**10.

4. Raubenheimer J: **An item selection procedure to maximise scale reliability and validity.** *SA J Ind Psychol* 2004, **30:**59-64.

5. Anderson JC, Gerbing DW: **An updated paradigm for scale development incorporating unidemensionality and its assessment.** *J Market Res* 1988, **25:**186-192.

6. Green SB, Lissitz RW, Mulaik SA: **Limitations of coefficient alpha as an index of test unidimensionality.** *Educ Psychol Meas* 1977, **37:**827-838.

7. Tavakol M, Dennick R: **Making sense of Cronbach's alpha.** *Int J Med Educ* 2011, **2:**53-55.

8. Mylonas K, Veligekas P, Gari A, Kontaxopoulou D: **Development and psychometric properties of the scale for self-consciousness assessment.** *Psychol Rep* 2012, **111:**233-252.

* A part of Discussion section is present in main text of the manuscript
